# The Stener Lesion

**Published:** 2012-07-18

**Authors:** Neilendu Kundu, Sofya Asfaw, Joshua Polster, Robert Lohman

**Affiliations:** ^a^Departments of General Surgery; ^b^Departments of Radiology; ^c^Plastic Surgery, Cleveland Clinic Foundation, Cleveland, Ohio

**Figure:**
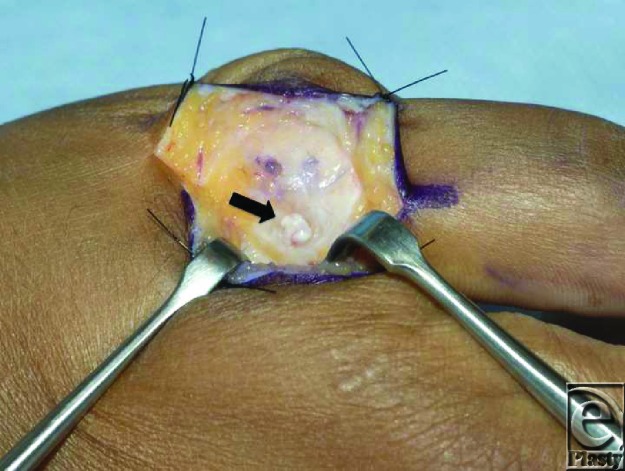


## DESCRIPTION

A 37-year-old man presented to our clinic after sustaining a low-speed motorcycle accident. He suffered minor facial abrasions and pain at his right thumb metacarpophalangeal (MCP) joint consistent with a gamekeeper injury. He was placed in a splint for several weeks; however, he had persistent pain and was thus evaluated with an MRI (magnetic resonance imaging) revealing a torn ulnar collateral ligament (UCL) with a Stener lesion.

## QUESTIONS

1. **What is a gamekeeper's thumb?**
2. **How was the disease entity coined?**
3. **What is the typical presentation and characteristic signs?**
4. **What is a Stener lesion?**
5. **What is the typical management of a gamekeeper's thumb?**

## DISCUSSION

Originally described by Campbell in 1955, the term gamekeeper's thumb describes chronic, thumb instability due to insufficiency of the UCL of the MCP joint.^1^ This was commonly observed in a nontraumatic setting among Scottish gamekeepers who would kill a wounded rabbit by holding the head in one hand and the buttocks (or rear legs) in the other and exerting a strong pull. While pulling, the neck was sharply extended in the thumb-forefinger cleft causing strain after continued repetition. A similar process occurs in the acute setting after trauma—typically a skier falling onto the ground while bracing hand with a ski pole—causing a valgus force on the thumb.^2^

Presentation can be somewhat varied from acute to chronic. However, symptoms are fairly characteristic including pain, ecchymosis of the thumb MCP, and weakness of the pinch grasp.

Most patients presenting with gamekeepers' thumbs can be managed nonoperatively with immobilization in the form of a long-arm thumb spica splint for several weeks. However, presence of a Stener lesion is a distinct, anatomic lesion that requires surgical correction. In cases of a complete, distal, thumb UCL tear, the aponeurosis of the adductor pollicis muscle can be interposed between the MCP of the joint and torn ligament. This aponeurosis maintains separation between the ruptured ends of the ligaments preventing healing.^3^ Thus, presence of a Stener lesion is an indication for surgical repair. This can be performed using a bone anchor to allow for reinsertion of the ligament.

Studies have not been able to arrive on a consensus regarding the utility of MRI in evaluating Stener lesions.^4^^-^6 We have found that MRI is of high utility in evaluation of patients with symptoms of gamekeeper's thumb.

## Figures and Tables

**Figure 1:**
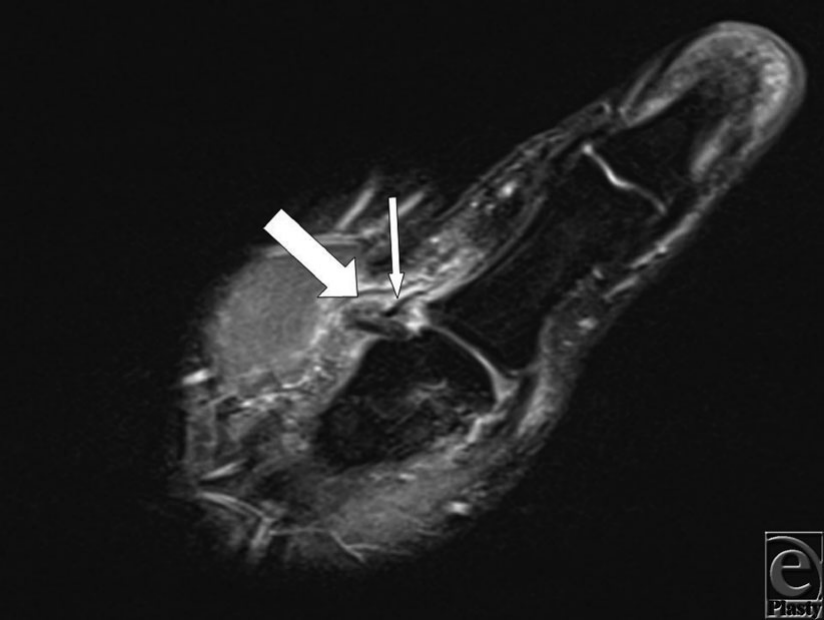
Coronal T2 weighted fat-suppressed MRI of the thumb demonstrating the ulnar collateral ligament (thick white arrow) subluxed ulnar to the adductor aponeurosis (thin white arrow).

**Figure 2:**
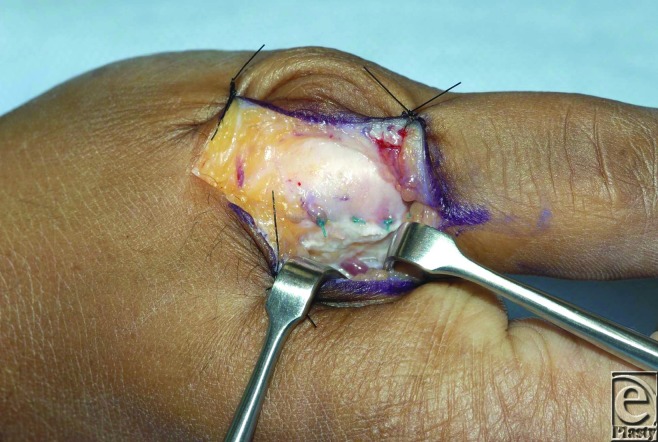
Appearance after repair using a bone anchor at the base of the proximal phalanx.
